# Arsenic trioxide-mediated suppression of miR-182-5p is associated with potent anti-oxidant effects through up-regulation of *SESN2*

**DOI:** 10.18632/oncotarget.24678

**Published:** 2018-03-23

**Authors:** Liang-Ting Lin, Shin-Yi Liu, Jyh-Der Leu, Chun-Yuan Chang, Shih-Hwa Chiou, Te-Chang Lee, Yi-Jang Lee

**Affiliations:** ^1^ Department of Biomedical Imaging and Radiological Sciences, National Yang-Ming University, Taipei, Taiwan; ^2^ Department of Radiation Oncology, MacKay Memorial Hospital, Taipei, Taiwan; ^3^ Division of Radiation Oncology, Taipei City Hospital Ren Ai Branch, Taipei, Taiwan; ^4^ Institute of Neuroscience, National Chengchi University, Taipei, Taiwan; ^5^ Department of Medical Research and Education, Taipei Veterans General Hospital, Taipei, Taiwan; ^6^ Institute of Clinical Medicine, School of Medicine, National Yang-Ming University, Taipei, Taiwan; ^7^ Institute of Pharmacology, National Yang-Ming University, Taipei, Taiwan; ^8^ Institute of Biomedical Sciences, Academia Sinica, Taipei, Taiwan; ^9^ Biophotonics and Molecular Imaging Research Center (BMIRC), National Yang-Ming University, Taipei, Taiwan; ^10^ Current address: Department of Health Technology and Informatics, The Hong Kong Polytechnic University, Hong Kong

**Keywords:** arsenic trioxide, sestrin 2, miR-182, oxidative stress, anti-oxidant effect

## Abstract

Arsenic trioxide (ATO) is a traditional Chinese medicine that can induce oxidative stress for treatment of cancer cells. However, ATO may generate anti-oxidative responses to compromise the cytotoxic effect, but the underlying mechanisms remain unclear. Here we found that ATO could inhibit miR-182-5p expression in patient-derived primary S1 glioblastoma (GBM) cells accompanied by up-regulation of Sestrin-2 (*SESN2*) mRNA, a known anti-oxidant molecule. This phenomenon was also detected in a U87MG glioma cell line, human lung adenocarcinoma H1299 cell line and A549 cell line. Pretreatment with a free radical scavenger N-acetylcysteine (NAC) reduced the oxidative stress induced by ATO. Concomitantly, ATO mediated suppression of miR-182-5p and enhancement of *SESN2* expression were also compromised. The MTT assay further showed that ATO induced cytotoxicity was enhanced by transfection of miR-182-5p mimics. Overexpression of miR-182-5p mimics significantly suppressed the expression of *SENS2* and a firefly luciferase reporter gene fused to 3’- untranslated region (UTR) of *SESN2* mRNA. Use of ribonucleoprotein immunoprecipitation (RNP-IP), ATO mediated suppression of miR-182-5p led to the stabilization of *SESN2* mRNA as a result of Argonaute-2 (AGO2) dependent gene silencing. Furthermore, high expression of miR-182-5p and low expression of *SESN2* mRNA tend to be associated with longer survival of glioma or lung cancer patients using public available gene expression datasets and online tools for prediction of clinical outcomes. Taken together, current data suggest that the miR-182-5p/SENS2 pathway is involved in ATO induced anti-oxidant responses, which may be important for the design of novel strategy for cancer treatment.

## INTRODUCTION

Although arsenic trioxide (ATO, As_2_O_3_) is a threat to human life because of its extreme toxicity, it has been widely used in various traditional Chinese medicine regimens. ATO is also regarded as an effective chemotherapeutic agent for treating acute promyelocytic leukemia (APL) and has been approved by the US Food and Drug Administration and State Drug Administration (SDA) in China [1, 2]. ATO also combines with all-trans retinoic acid (ATRA), a first-line agent for APL for treatment of some types of leukemia [3]. The therapeutic efficacy of ATO was also found in human lymphoma, chronic myelogenous leukemia (CML), gastric cancer cells, hepatocellular carcinoma cells, neuroblastoma cells, head and neck cancer cells, lung cancer, and esophageal cancer cells [2, 4–8]. Additionally, ATO has been reported to enhance the sensitivity of glioblastoma (GBM) to a Myc inhibitor [9]. Although the molecular mechanisms remain unclear, the ATO induced oxidative stress is believed to cause defects in genomic stability, epigenetic modulation, gene expression, cell cycle progression, and in triggering the mitochondrial pathway of apoptosis [10, 11]. On the other hand, ATO can induce antioxidant activities that may compromise the drug’s effects [12], but the detailed mechanisms remain to be addressed.

MicroRNAs (miRNAs) are small non-protein-coding RNAs originally transcribed from introns or exons of certain genes [13]. RNA polymerase II is responsible for transcription of primary miRNA (pri-miRNA) that forms a hairpin structure containing miRNA sequence, yet RNA editing, modification, and Argonaute (AGO) loading are additional levels of miRNA biogenesis [14, 15]. Active miRNAs about ~22 nucleotides in length after processing of pri-miRNA by ribonuclease III (RNase III) Drosha and Dicer [16, 17], while a dicer-independent pathway was also reported [18]. It has been well established that miRNAs help regulate gene expression and various biological processes through post-transcriptional mechanisms, especially by recognizing the 3’-untranslated region (3’-UTR) of mRNA [19]. To date, more than 1,000 human miRNAs have been found to regulate one-third of all human genes [20]. The AGO family proteins mediate miRNA-binding to mRNA and form the catalytic component of RNA-induced silencing complex (RISC) that targets specific mRNAs and leads to their decay or translational repression [21–23]. Several lines of evidence have shown that hsa-miR-182-5p (abbreviated as miR-182-5p) expression is associated with human malignancies, including hepatoma, glioma, ovarian carcinoma, and bladder cancer [24–27]. However, miR-182-5p was also found to suppress the growth or proliferation of lung cancer and gastric cancer *in vitro* [28, 29]. A recent study showed that high intracellular levels of reactive oxygen species (ROS) induced the expression of miR-182-5p, which results in different cell fates in fallopian tube secretory epithelial cells containing wild-type p53 versus mutant one [30]. Whether the expression of miR-182-5p is involved in ATO induced oxidative responses remains unclear.

*SESN2* is an anti-oxidant protein evolutionally conserved in various species and is inducible by oxidative, genotoxic and energetic stress [31]. *SESN2* can reduce cysteine formed a sulfinic acid of peroxiredoxins (Prxs), which is unreducible by the thioredoxin system [32]. *SESN2* is a p53 target gene that mediates the p53 response to oxidative stress and genotoxic stress in a cell-protective manner [33]. However, p53-independent induction of *SESN2* has also been reported in hypoxic cells and glucose-starved cells [34, 35]. SESN2 protein also prevents cells from energetic stress-induced death through the integration of the protein kinase B (Akt) and mammalian tolerance of rapamycin (mTOR) signaling pathway [36]. Ionizing radiation has been reported to induce *SESN2* expression in glioblastoma cells, but silencing of *SESN2* not only increases intracellular oxidative stress but also sensitizes cells to ionizing radiation [37]. It is still unclear whether *SESN2* is also involved in ATO-induced anti-oxidant responses.

Oxidative stress plays an important role in tumor development as well as anticancer therapy. In this study, we explored whether miR-182-5p and *SESN2* are involved in ATO-induced oxidative responses. Our results suggest that miR-182-5p is important for modulating ATO-mediated *SESN2* expression and cell death.

## RESULTS

### Bioinformatics analysis implicates the involvement of miR-182-5p in ATO-associated responses

In 2013, Hara-Yamamura et al. used a cDNA microarray to investigate low (0.05μM) to high (40μM) concentration of ATO-induced alterations in gene expression of HepG2 hepatocellular carcinoma cells (see Materials and Methods, https://www.ncbi.nlm.nih.gov/geo/query/acc.cgi?acc=GSE48441). We utilized a hierarchical clustering method (data not shown), and gene ontology analysis showed that moderate concentration of ATO (6 μM) tended to increase the expression of genes related to protective responses compared to treatment with high-dose ATO (40 μM). For instance, ATO caused gene expressions were mainly associated with response to external stress and stimulus compared to another category of gene ontology (GO) analysis (Supplementary Figure 1A). To narrow the spectrum of regulated genes, we performed GSEA, a functional enrichment analysis used to evaluate microarray data based on the prior gene sets with known biological functions [38]. Interestingly, use of miRNA-associated gene set (c3.mir.V5.2.symbols.gmt, see Materials and Methods) revealed that miR-182-5p-associated genes were significantly enriched (maximum ES values of 0.01 and 0.042 for up-regulated genes and down-regulated genes, respectively. Supplementary Figure 1B).

### ATO induces oxidative stress and SESN2 expression via suppression of miR-182-5p

**Figure 1:**
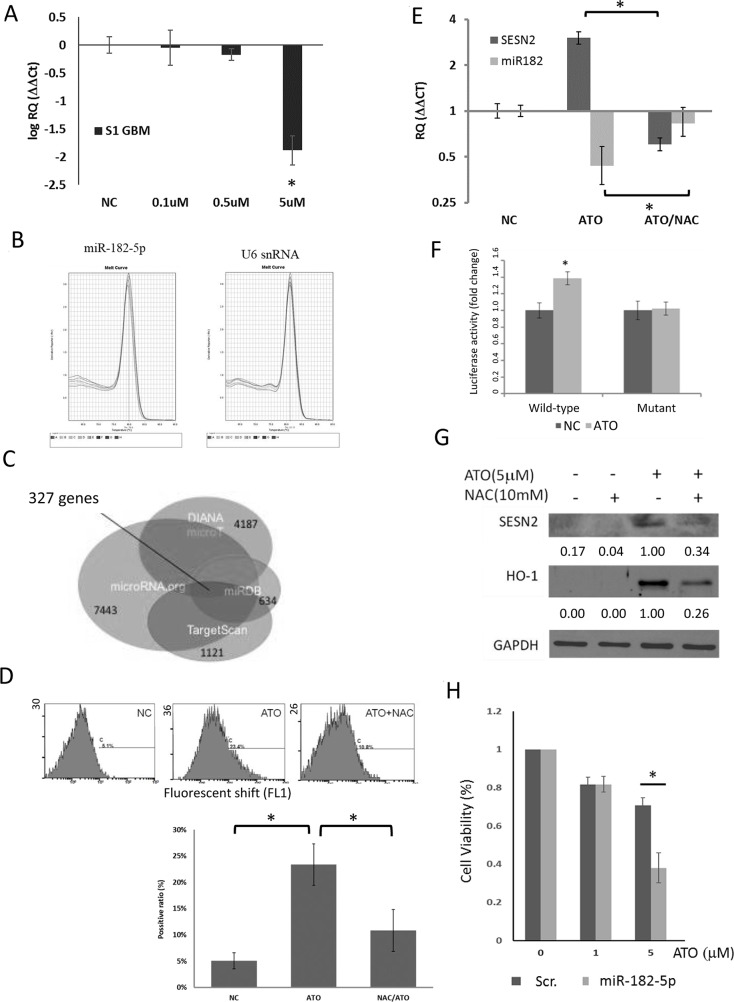
Effect of ATO on miR-182-5p expression in S1 GBM cells **(A)** Dose-dependent suppression of miR-182-5p expression by ATO (n=4). **(B)** The melting curves of miR-182-5p and U6 snRNA during the qRT-PCR. **(C)** The Venn diagram involved four databases that predict putative mRNA targeted by miR-182-5p. The number of each colored circle was the amount of predicted mRNAs. There were 327 predicted mRNAs repeatedly presented in these four databases. **(D)** Flow cytometric analysis of ATO-induced oxidative stress. **(E)** NAC rescued ATO-mediated suppression of miR-182 expression. **(F)** Reporter gene assay results showed that ATO suppressed miR-182 targeting of the 3’UTR of the *SESN2* gene. **(G)** Suppression of ATO-mediated induction of *SESN2* by NAC. **(H)** The results of M TT assay showed that transduction with a miR-182 mimic enhanced the cytotoxic effects of ATO. ^*^: p<0.05.

We previously found that ATO could induce oxidative stress in a U87MG glioma cell line [39]. To investigate whether ATO could also influence the expression of miR-182-5p, The qRT-PCR analysis was used to determine the expression of miR-182-5p in cells exposed to ATO. First, patient-derived human S1 primary GBM cells related to S1R1 recurrent GBM reported before were used for the treatment of different concentrations of ATO [40]. Interestingly, miR-182-5p could be significantly inhibited by 5μM ATO (Figure 1A). The melting curves performed in amplification of miR-182-5p and U6 snRNA control demonstrated no nonspecific products were visualized in qRT-PCR (Figure 1B). ATO suppressed miR-182-5p was also detected in U87MG cells, A549 cells and H1299 cells using the same concentration (Supplementary Figure 2).

Since robust online databases have developed microRNA binding prediction based on diversified algorithms, we individually acquired the list of predicted mRNA targets of miR-182-5p from four databases including microRNA.org, TargetScan V6.2, miRDB V5.0, and DIANA microT CDS. We then used mirDIP online tool (cut-off value set as ≥ 0.01) to conclude the overlapped mRNA targets among the four databases. Three hundred and twenty-seven overlapped mRNA targeted by miR-182-5p were displayed in a Venn diagram, which is used for exhibiting logical relations among a collection of different databases (Figure 1C). Among them, we noticed that *SESN2* gene was one of the miR-182-5p targets because the predicted binding sequence of miR-182-5p was found to be matched with the 3’-UTR of *SESN2* mRNA from +1020 to +1027 bps. We have previously shown that silencing of *SESN2* led to increase of oxidative stress, suggesting that *SESN2* gene plays an anti-oxidative role [37]. Although the ranks of *SESN2* targeted by miR-182-5p was moderate (Table 1), we still decided to investigate if ATO suppressed miR-182-5p would correlate to *SESN2* expression and associated oxidative stress response. Use of the DCFDA and flow cytometric analysis, ATO was demonstrated to induce oxidative stress, which could be alleviated by treatment with the free radical scavenger NAC in S1 GBM cells (Figure 1D) [41]. ATO-mediated suppression of miR-182-5p led to up-regulation of *SESN2* mRNA, whereas NAC reversed this phenomenon (Figure 1E). ATO also increased the luciferase activity of fLuc reporter genes fused to the wild-type 3 ’UTR, but not a mutant 3 ’UTR, of *SESN2* mRNA (Figure 1F). Furthermore, SESN2 protein expression was also induced by ATO but was reduced by NAC pretreatment (Figure 1G). Heme oxygenase 1 (HO-1) was used as a control that can be induced by ATO as a result of oxidative stress [42, 43]. ATO induced *SESN2* was also detected in U87MG cells, A549 cells and H1299 cells that have exhibited reduced miR-182-5p mentioned above (Supplementary Figure 3). These findings suggested that ATO-mediated induction of oxidative stress suppressed miR-182-5p, leading to the up-regulation of *SESN2*. A cytotoxicity assay showed that transfection of a miR-182 mimic into S1 GBM cells substantially enhanced the cytotoxic effect of ATO at higher concentrations (Figure 1H). These data suggest that ATO-mediated suppression of miR-182 protects cells from oxidative stress by inducing the expression of the anti-oxidative molecule *SESN2*.

**Table 1:** Ranking of SESN2 and TP53INP1 regulated by miRNA-182 in four different databases

SESN2	DIANA-microT^*^	microRNA.org^†^	TargetScan^§^	miRDB^¶^
Ranking	2022/4187^*^	2595/7443	395/1121	241/634
Percentage	48.29%	34.86%	35.23%	38.01%
TP53INP1	DIANA-microT	microRNA.org	TargetScan	miRDB
Ranking	30/4187	1305/7443	179/1121	139/634
Percentage	0.72%	17.53%	15.97%	21.92%

^*^The threshold for gating the results was set as 0.5.

^†^ The last update of the database was Nov. 1st, 2010.

^§^ TargetScan 6.2 (release on June, 2012) was used for the analysis.

^¶^ The latest modification for the database was May 3rd, 2016.

### Effects of miR-182-5p on SESN2 expression

**Figure 2:**
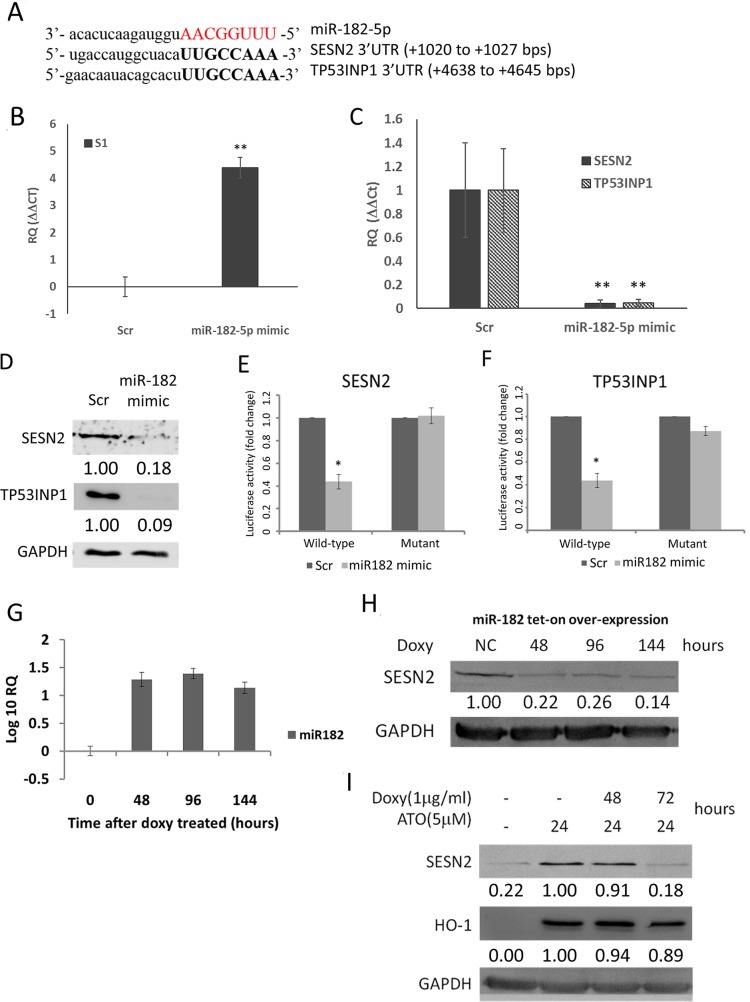
MiR-182-5p suppressed the expression of *SESN2* and *TP53INP1* **(A)** Sequence complementary between miR-182-5p and 3’-UTR of *SESN2* and *TP53INP1*. **(B)** Confirmation of miR-182-5p mimic transduction into GBM cells by qRT-PCR. **(C)** *SESN2* mRNA and *TP53INP1* mRNA expression was inhibited following transduction of the miR-182-5p mimic. **(D)** SESN2 and TP53INP1 protein expression were inhibited following transduction of the miR-182-5p mimic. **(E)** and **(F)** Reporter gene assay results demonstrating that the miR-182-5p mimic targets the 3’UTRs of *SESN2* mRNA and *TP53INP1* mRNA. **(G)** Tetracycline-induced miR-182-5p expression in H1299 cells transduced with a *tet-on*-miRNA system. **(H)** Suppression of SENS2 protein expression following *tet-on* induction of miR-182-5p expression. **(I)** Suppression of ATO induced SENS2 expression, but not HO-1 expression, following *tet-on* induction of miR-182-5p expression. ^*^: p<0.05.

We next examined whether miR-182-5p would directly inhibit *SESN2*. *TP53INP1*, another anti-oxidant gene known to be directly regulated by miR-182-5p [44], was co-examined with *SESN2* after cells were transfected with miR-182-5p mimics. The estimated rankings of *TP53INP1* targeted by miR-182-5p were also performed in four different databases as described above (Table 1). First, we showed that the 3’-UTR of *SESN2* and *TP53INP1* shared identical targeting sequences recognized by miR-182-5p (Figure 2A). A miR-182-5p mimic was subsequently transfected into S1 GBM cells and confirmed using qRT-PCR (Figure 2B). The transfection led to significant suppression of *SESN2* and *TP53INP1* mRNA at similar levels (Figure 2C) as well as suppression of protein expression (Figure 2D). Furthermore, the miR-182-5p mimic suppressed the luciferase activity of fLuc reporter genes fused to the wild-type 3 ’UTRs, but not mutant forms of the 3 ’UTRs, of *SESN2* and *TP53INP1* mRNA (Figure 2E and 2F).

To examine if over-expression of miR-182-5p could also regulate *SESN2* and *TP53INP1* in different cell type, we introduced a tetracycline-inducible (*tet*-on) miR-182-5p expression system to H1299 lung cancer cells to express miR-182-5p. The qRT-PCR data showed that doxycycline-induced miR-182-5p expression was sustained for up to 144 hours in H1299 cells (Figure 2G). Overexpression of miR-182-5p also markedly suppressed *SESN2* expression over the same period (Figure 2H). Nevertheless, the basal *TP53INP1* level was too low to be detected in H1299 cells (data not shown). In contrast to *SESN2*, HO-1, which is not a target of miR-182-5p, was significantly induced by ATO but not suppressed by overexpression of miR-182-5p (Figure 2I). These results support that miR-182-5p would play an important role in regulating *SESN2* expression.

### ATO-mediated suppression of miR-182-5p recruited AGO2 to the mRNA, leading to Degradation

**Figure 3:**
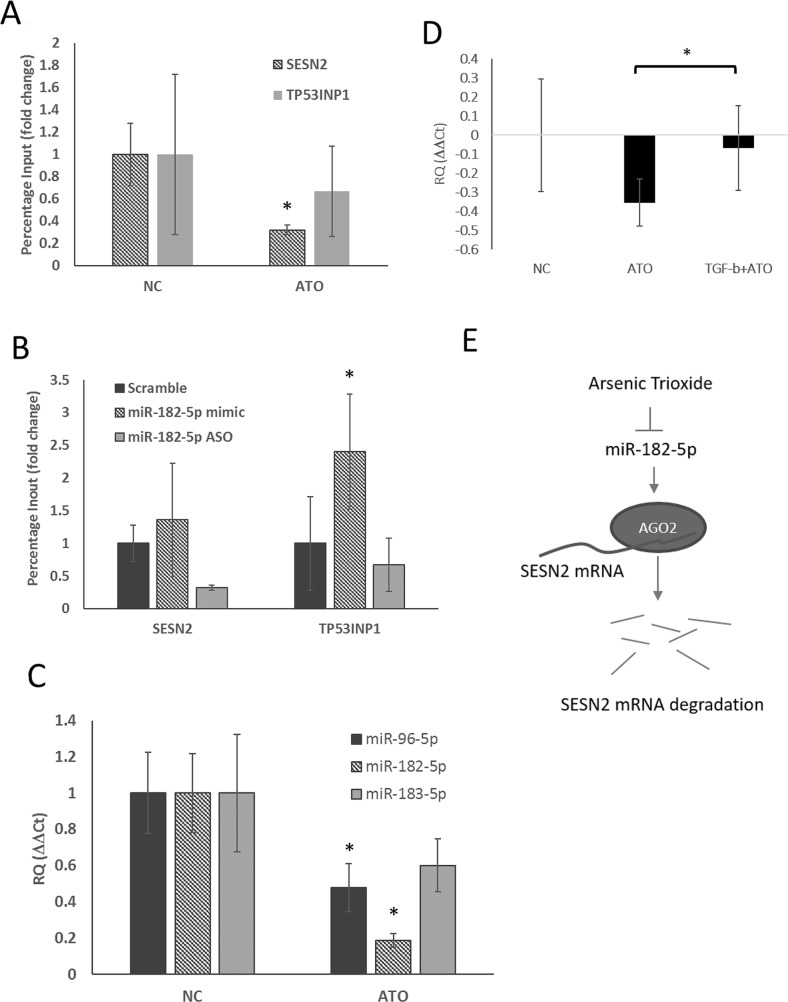
Suppression of miR-182-5p mediated binding of AGO2 to *SESN2* mRNA following treatment with ATO **(A)** RNP-IP was used to evaluate the binding ability of AGO2 to SENS2 mRNA and *TP53INP1* mRNA before and after ATO treatment (see text) (n=3). **(B)** The influence of manipulating miR-182-5p expression on AGO2 binding to *SESN2* mRNA and *TP53INP1* mRNA. **(C)** Suppression of miR-183/96/182 gene cluster expression by ATO. **(D)** ATO-mediated suppression of miR-182-5p expression was rescued by TGF-β. **(E)** A putative model shows ATO inhibiting the binding of AGO2 to mRNA via suppression of miR-182-5p expression. ^*^: p<0.05.

**Figure 4:**
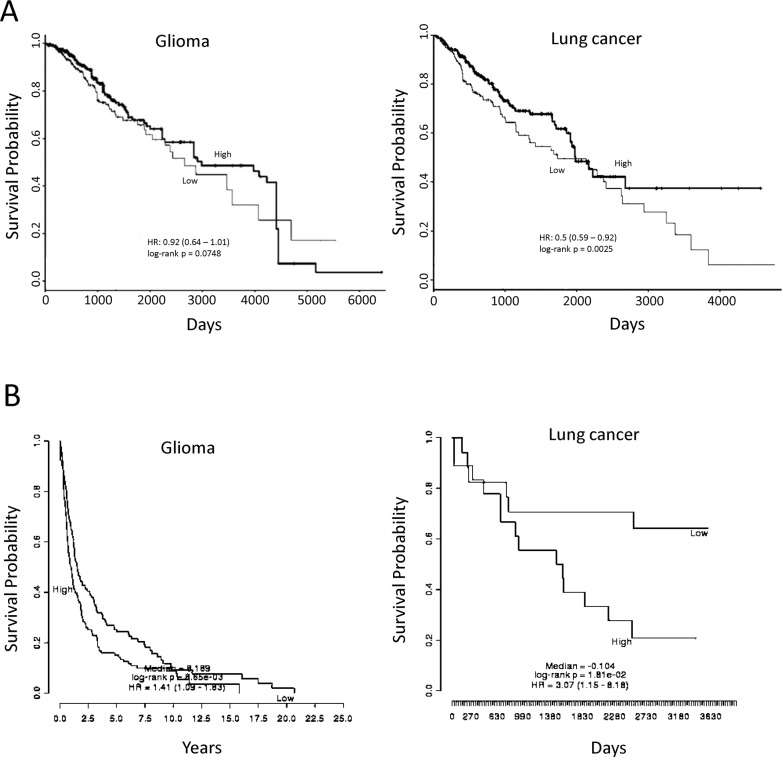
Association of miR-182-5p and *SESN2* expression with overall survival using publicly available datasets **(A)** Comparison of high and low miR-182-5p expressive levels on overall survivals of glioma (TCGA Brain Lower Grade Glioma data) and lung cancer patient (TCGA Lung Squamous Cell Carcinoma data); **(B)** Comparison of high and low *SESN2* mRNA expressive levels on overall survivals of glioma (accession No. GSE16011) and lung cancer patient (accession No. GSE 11969). A p-value below 0.05 represented a significant difference.

To determine how ATO mediates miR-182-5p-associated gene silencing, we investigated whether the binding of the miRNA-guided protein AGO2 to *SESN2* and *TP53INP1* mRNA was regulated by ATO. Using RNP-IP, we showed that ATO treatment significantly decreased the quantity of AGO2 bound to *SESN2* mRNA in S1 GBM cells, suggesting that AGO2-mediated mRNA degradation was suppressed by ATO (Figure 3A). The binding of AGO2 to *TP53INP1* mRNA was also slightly, but not significantly, reduced; thus, the results could have been due to sample variation. Because ATO suppressed *miR-182-5p*, we next examined whether manipulating miR-182-5p expression affected the binding of AGO2 to *SESN2* and *TP53INP1* mRNA. The results showed that transfection of the miR-182-5p mimic weakly increased the binding of AGO2 to *SENS2* mRNA and *TP53INP1* mRNA, whereas transfection of an Anti-Sense Oligonucleotide for miR-182-5p (miR-182-5p ASO) significantly decreased the binding of AGO2 to *SESN2* mRNA compared to scramble-transduced cells (Figure 4B). However, miR-182-5p ASO did not significantly inhibit the binding of AGO2 to *TP53INP1* mRNA (Figure 3B). Additionally, ATO suppressed the expression of not only miR-182-5p but also miR-96-5p and miR-183-5p, which are derived from the same transcript as miR-182-5p (Figure 3C). These results suggest that ATO suppresses the transcription of the miR-182/183/96 gene cluster as a whole. Three separate transformation growth factor β (TGF-β)-mediated Smad2/3-responding elements (SREs) were found located approximately −2000 to −3500 bp upstream of the transcription start site of the miR-182/183/96 gene cluster. Interestingly, ATO-mediated suppression of the miR-182/183/96 gene cluster tended to be restored by pretreating cells with TGF-β (Figure 3D). A model showing the pathway for ATO-mediated suppression of the miR-182-5p gene cluster and regulation of AGO2 for stabilizing *SESN2* mRNA was illustrated (Figure 3E).

### The survival implication for miR-182-5p and SESN2

Because ATO could suppress miR-182-5p and lead to up-regulation of *SESN2*, it is interesting to investigate whether these genes would be associated with overall survival of patients. PROGmiR [45] and PRECOG [46] were two online tools used for assessment of specific microRNA and mRNA on the survivals of different cancer types, respectively. The datasets were retrieved from GEO and The Cancer Genome Atlas (TCGA) to query whether the expression of specific microRNA or mRNA would be prognostic for patient survival rate. We found that high expression of miR-182-5p represented longer survival in lung cancer (Figure 4A). A similar trend was also found in glioma, but the significance was margin (Figure 4A). For *SESN2* gene, the low expression of *SESN2* showed significantly longer survival in both glioma and lung cancer (Figure 4B). These preliminary analyses using public available datasets implies that high expression of miR-182-5p and low expression of *SESN2* may lead to higher survival rates.

## DISCUSSION

Although oxidative stress causes cytotoxicity, this insult can also induce anti-oxidant responses in cells. These self-protective responses are important for cell survival but in turn, create a therapeutic conflict when using radiochemotherapy for cancer treatment. Arsenic is a well-known poison that reacts with enzymes and corresponding ligands with active sulfur groups [47]. Despite this, arsenic has been widely used in traditional Chinese medicine and is also approved by the FDA for treatment of APL [1]. Because ATO can partially penetrate through the blood-brain barrier via general intravenous infusion or oral administration [48, 49], concurrent therapy has combined ATO and radiotherapy in the preclinical and clinical trial [50, 51]. A recent phase II trial has also been reported by combining ATO and temozolomide (TMZ) with radiotherapy for malignant gliomas [52]. However, patients showing resistance to ATO have posed a significant clinical problem, and strategies for addressing this problem are not yet available [53]. Here, we showed that moderate doses of ATO significantly suppress miR-182 expression, leading to up-regulation of the anti-oxidant molecule *SESN2* in GBM cells. Restoration of miR-182 expression significantly enhanced the cytotoxicity of ATO, suggesting that ATO controls the miR-182 pathway to modulate anti-oxidant responses.

Different miRNA species have been reported to respond to oxidative stress *in vitro* and *in vivo* [54, 55]. Although ATO is known to induce oxidative stress, little is known regarding whether this is the primary factor suppressing miR-182-5p and inducing *SESN2*. Using the ROS scavenger NAC in ATO-treated cells demonstrated that the effect of ATO on *SESN2* and miR-182 expression is directly related to oxidative stress. However, a recent report showed that ROS generated by hydrogen peroxide-induced miR-182-5p expression via the Wnt/β-catenin signaling pathway in high-grade serous ovarian carcinoma [30]. Thus, the biological function of oxidative stress-induced miR-182 expression should be further investigated.

The phosphoinositide 3-kinase (PI3K)/AKT and p38-dependent pathways have been associated with the molecular mechanism underlying the effectiveness of ATO treatment in leukemia [56, 57]. Additionally, ATO could influence miRNA-dependent methods of suppressing leukemic cancer cell survival [58–60]. The tumor suppressive functions of ATO have also been associated with miRNAs, such as miR-125b in glioma cells and miR-27a in breast cancer cells [61, 62]. Here we found that miR-182-5p plays an important role in regulating the expression of anti-oxidant related genes to protect against ATO. ATO-mediated suppression of miR-182-5p was demonstrated in both GBM cells and lung cancer cells, complementing the original findings based on HepG2 cells. The cell lines used here (U87MG, A549, and H1299) have been reported to be treated with ATO for investigation of cell survival and apoptosis [63–65]. ATO induced down-regulation of miR-182-5p accompanied by up-regulation of *SESN2* in these cell lines suggests that ATO-mediated regulation of miR-182-5p expression may be an important therapeutic property.

MiR-182-5p expression is up-regulated in hepatocellular carcinoma, and the miRNA has been shown to function as an oncogene by targeting various genes, including *SESN2* and *TP53INP1*, related to cancer [66]. Thus, suppression of miR-182-5p by ATO may account for the therapeutic efficacy of this compound in this cancer. However, miR-182-5p is regarded to have tumor-suppressor functions in glioblastoma and osteosarcoma [67, 68]. Our data also showed that overexpression of a miR-182-5p mimic enhanced ATO-induced cytotoxicity in GBM, suggesting that miR-182-5p plays a tumor-suppressive role that is consistent with previous reports [67–69].

The miRNA-mediated suppression of mRNA translation is dependent on RISC function [70]. As a catalytic component of RISC, AGO2 binds to the 5’ and 3’ ends of mature miRNA, leading to degradation [71]. Here, we showed that the binding of AGO2 to *SESN2* mRNA was attenuated by ATO as well as by transfection with anti-miR-182-5p (ASO), suggesting that ATO directly suppresses miR-182-5p to control the up-regulation of *SESN2*. The binding of AGO2 to *TP53INP1* in ATO-treated cells was also attenuated, but the reduction was not statistically significant. The lack of significance likely resulted from the wide margin of error that was produced when analyzing *TP53INP1* mRNA expression (Figure 4A). However, miR-182-5p ASO showed no significant inhibition of AGO2 binding to *TP53INP1* mRNA, suggesting that *TP53INP1* mRNA may be targeted by miR-182 through the aid of a mRNA-binding protein other than AGO2. Additionally, ATO down-regulated the expression of miR-96 and miR-183 in addition to miR-182, and all three miRNAs are located on the same gene cluster. Interestingly, the TGF-β signaling pathway, which transactivates the miR-96/miR-182/miR-183 gene cluster [72], compromised the effects of ATO-mediated suppression of miR-182-5p. Because TGF-β is essential for tumor progression, the effect of ATO on this pathway should be further investigated for tumor control.

The potent clinical outcomes of miR-182-5p and *SESN2* gene expression were also evaluated using online tools. It appeared that the analytic results using PROGmiR and PRECOG partially agreed our findings because high expression of miR-182-5p and low expression of *SESN2* contributed to longer survivals in glioma and lung cancers. However, we could not exclude that results from other datasets and tools might be different. Besides, whether ATO induced anti-oxidant effects would substantially influence the overall survival is completely blurred. It is surely of interest to be investigated in the future.

In summary, down-regulation of miR-182-5p plays an important role in the anti-oxidant effects resulting from ATO treatment. miR-182-5p targeting of *SESN2* and/or *TP53INP1* may be directly responsible for this phenomenon. Use of moderate concentrations of this chemotherapeutic agent is essential to avoid complications affecting normal tissues, but the therapeutic effect may be compromised as a result. Because moderate doses of ATO also alter gene expression, obtaining a better understanding of the signaling pathways affected by the compound would be beneficial for designing new therapeutic strategies. Ablation of miR-182 during ATO treatment may enhance the therapeutic efficacy of this compound.

## MATERIALS AND METHODS

### Cell culture, transfection, and chemical treatments

The patient-derived S1 GBM primary cells were cultured in Dulbecco’s Modified Eagle’s Media (DMEM, Life Technologies Inc., Carlsbad, CA, USA) supplemented with 10% fetal bovine serum (HyClone Laboratories Inc., South Logan, UT, USA), 100 units/mL penicillin, and 100 μg/mL streptomycin (Life Technologies Inc., Carlsbad, CA, USA) under standard culture conditions (37°C, 95 % humidified air and 5% CO_2_) [40]. Human U87MG glioma cell line (American Type Culture and Collection, ATCC^®^ HTB-14^TM^) was cultured in Modified Eagle’s Media (MEM, Life Technologies Inc., Carlsbad, CA, USA), and human lung adenocarcinoma A549 cell line and H1299 cell line were cultured in RPMI media 1640 with supplements described above. Subcultures were generated with 0.25% trypsin-EDTA (Sigma-Aldrich Co., St. Louis, MI, USA) for a two-day interval. Plasmid DNA transfection was performed using jetPEI DNA transfection reagent (Cat. #: 101-10N, Polyplus, Huntingdon, UK). The miR182-5p mirVana inhibitor (Cat. #: 4464084, Thermo Fisher Scientific Inc., Waltham, MA, USA) and miRVana mimic (Cat. #: 4464066, Thermo Fisher Scientific Inc., Waltham, MA, USA) were prepared in 10nM stock solution and transfected by INTERFERin® siRNA/miRNA transfection reagent (Cat. #: 409-10 Polyplus, Huntingdon, UK) following the manufacturer’s instructions. ATO (Cat. #:202673, Sigma Aldrich Co., St. Louis, MI, USA) was dissolved in 1 N NaOH at a concentration of 100 mM as a stock solution. N-acetyl cysteine (NAC) was prepared as a 1 M stock solution in sterilized water.

### Bioinformatic analysis of gene expression

A gene expression array (GSE48441) including data from ATO-treated Hep2 hepatocellular carcinoma cells was obtained from Gene Expression Omnibus (GEO, https://www.ncbi.nlm.nih.gov/geo/) [73]. The genetic network connection was performed using Ingenuity Pathway Analysis (IPA) software (Qiagen, Hilden, Germany). The cutoff used a log2 ratio to compile a list of genes for further analysis. Genetic function and gene enrichment analysis was performed using DAVID Bioinformatics Resources (http://david.abcc.ncifcrf.gov/). Gene ontology (GO) terms were further summarized using REVIGO (http://revigo.irb.hr/). For gene set enrichment analysis (GSEA), the Java-based GSEA software was used (Broad Institute, Cambridge, MA, USA). The gene expressive profile was converted into the appropriate format for input (gct file) with the elimination of only marginally impacted genes (less than a 5-fold change) for GSEA. Initial calculations were made using MSigDB-integrated gene sets (c3.mir.V5.2.symbols.gmt). The genes predicted to be targeted by miR-182-5p in all four microRNA prediction databases (DIANA, microRNA.org, miRDB, and TargetScan) were included in the gene set for further computing.

### Plasmid construction

For reporter-based luciferase activity assays, we flanked a *SESN2* 3’UTR fragment with the primers *SESN2*_s: 5’-gggAgAATTCTgTTCTCCCAg-3’ and *SESN2*_as: 5’-TgCACTTgAACACTggATACC-3’ using Phusion Hot Start II PCR (Cat #: F549S, Thermo Fisher Scientific Inc., Waltham, MA). The amplicon was digested with XbaI (Cat #: R0145S, New England Biolabs Inc., Ipswich, MA) for a 637-bp fragment which was subsequently inserted into pGL4.10-Luc2 vector (Promega, Madison, WI). The cytomegalovirus (CMV) promoter was placed into the pGL4.10-Luc-*SESN2* 3’UTR vector (Supplementary Figure 4) to promote gene transcription, and form pCL-*SESN2*_3’UTR construct for reporter assay. To manipulate the miR-182-5p-binding site, we replaced the predicted binding element, TTgCCAAA, with a restriction enzyme XbaI site, TCTAgA, using the primer *SESN2*_Muta: 5’-TgACCATggCTACATCTAgACCTCTgACTgCCACAgCT-3’ and *SESN2*_as.

### Target predication database analysis

Four databases were used in this study including TargetScan V6.2 (http://www.targetscan.org/vert_61/, released on June, 2012) [74], microRNA.org (http://www.microrna.org/microrna/home.do, released on November, 2010) [75], DIANA microT CDS V5 (http://www.microrna.gr/microT-CDS, with threshold set as 0.5) [76, 77], and miRDB V5.0 (http://mirdb.org/) [78]. To bring out the overlapped genes predicted from all four databases, mirDIP developed from the University of Toronto was applied (http://ophid.utoronto.ca/mirDIP/index.jsp) [79].

### RNA isolation and real-time quantitative PCR

Monolayer-cultured cells were homogenized with TRIzol (Cat #:15596026, Thermo Fisher Scientific Inc., Waltham, MA), and RNA was purified using a Direct-zol RNA miniprep kit (Cat. #: R2050, Zymo Research Corp., Irvine, CA) following the manufacturer’s instructions. Reverse transcription was carried out using SuperScript III reverse transcriptase (Cat #.:18080093, Thermo Fisher Scientific Inc., Waltham, MA) with the following gene-specific primers: U6 stemloop, gTCgTATCCAgTgCAg ggTCCgAggTATTCgCACTggATACgACAAAATATgg AAC and miR-182 stemloop, gTCgTATCC AgTgCAgggTCCgAggTATTCgCACTggATACgACAgTgTg. To normalize miR-182-5p expression, U6 ncRNA was used as an internal control. For coding genes, total RNA was reverse transcribed using oligo dT primers. Quantitative PCR was performed using SYBR FAST Reagent (Cat #. KR0389, Kapa Biosystems Inc., Woburn, MA), and the results were analyzed on a StepOne Plus machine using the manufacturer’s software (Life Technologies Corp., Carlsbad, CA). The primers used in this study were listed (Table 2). Expression of the target gene was normalized to β-actin and U6 snRNA as endogenous and experimental controls, respectively, using the ΔΔCt method.

**Table 2:** The information of primers used for amplification of different miRNA and mRNA targets

Primers	Sequence
Universal reverse primer	CCAgTgCAgggTCCgAggT
miR96_stemloopRT	gTCgTATCCAgTgCAgggTCCgAggTATTCgCACTggATACgACAgCAAA
miR182_stemloopRT	gTCgTATCCAgTgCAgggTCCgAggTATTCgCACTggATACgACAgTgTg
miR183_stemloopRT	gTCgTATCCAgTgCAgggTCCgAggTATTCgCACTggATACgACAgTgAA
U6_stemloopRT	gTCgTATCCAgTgCAgggTCCgAggTATTCgCACTggATACgACAAAATATggAAC
miR96_qPCR F	gCCgCTTTggCACTAgCACATT
miR182_qPCR_F	TgCggTTTggCAATggTAgAAC
miR183_qPCR F	gCCgCTTATggCACTggTAgAA
U6_qPCR_loopF	TgCgggTgCTCgCTTCggCAgC
SESN2_s	AAgACCCTACTTTCggA
SESN2_as	CTgCCTggAACTTCTCAT
TP53INP1UTR_s	CAAggAAgAgTATCTTCATTATTCT
TP53INP1UTR_as	TgCTCTgAgTgAAgTTAAAggACACTTTATTT

### Western blot analysis

Protein lysates were prepared with RIPA buffer (Thermo Scientific Inc., Waltham, MA, USA) containing 1% protease inhibitor. An equal amount of total protein was subjected to SDS/PAGE and then transferred onto a nitrocellulose membrane (PALL Co., Port Washington, NY, USA). The blots were incubated with blocking buffer (TBS-T with 4% skim milk) at room temperature for 1 hour and then hybridized with primary antibodies overnight at 4°C followed by the horseradish peroxidase-conjugated secondary antibody incubation. Signals were illuminated using Enhanced Chemiluminescence reagent (Bio-Rad Laboratories Inc., Hercules, CA, USA) and recorded on an Image-Quant LAS-4000 imaging system (GE Healthcare, Chicago, IL, USA). The antibodies used in this study included anti-*SESN2* antibody (Abnova Inc., Taipei City, Taiwan), anti-heme oxygenase 1 (HO-1) antibody (Santz Cruz Biotechnology Inc., Dallas, TX, USA), anti-*TP53INP1* antibody (Sigma-Aldrich Co., St. Louis, MI, USA), and anti-glyceraldehyde phosphate dehydrogenase (GAPDH) antibody (GeneTex Inc., Alton Pkwy Irvine, CA, USA).

### Oxidative stress analysis

Reactive oxygen species (ROS) were identified using the 2’,7’ –dichlorofluorescein diacetate (DCFDA) and flow-cytometry analysis as reported previously [80]. DCFDA is a cell-permeable fluorogenic probe commonly used for detecting the status of cellular redox directly. In brief, S1 GBM cells were seeded 24 hours before experimental manipulation. The cells were pretreated with ten mM NAC for 4 hours or left untreated before ATO treatment. After incubation overnight, the cells were washed twice with pre-warmed PBS and detached from the culture dishes using 0.3% trypsin. The cells were collected by centrifugation and were washed twice with pre-warmed PBS, followed by 5 μM 2’,7’ –dichlorofluorescein diacetate (Invitrogen Inc., Carlsbad, CA, USA) incubation for 30 minutes. Then, the staining agent was replaced with culture medium, and the cells were placed back in an incubator for a 30-minute recovery. The cells were then washed with chilled PBS, detached using a scraper, passed through filter mesh, and subjected to flow cytometry analysis (FC500, Beckman Coulter Inc., Brea, CA, USA).

### Cytotoxicity assay

Cells were seeded in 96-well plates (3000 cells per well) with complete growth medium overnight, and the substances being tested were added to the medium at the indicated concentrations. Cell viability was assayed following incubation with 0.5 mg/ml 3-(4,5-cimethylthiazol-2-yl)-2,5-diphenyl tetrazolium bromide (MTT, Sigma-Aldrich Co., St. Louis, MI, USA) for 2 hours. The resulting formazan crystals were dissolved with DMSO, and the plates were placed in an ELISA plate reader (Tecan, Thermo Scientific Inc., Waltham, MA, USA) to measure light absorbance at a wavelength of 570 nm. The relative cytotoxic effects were then calculated from optic density (O.D.) readouts normalized to the control group.

### Ribonucleoprotein immunoprecipitation (RNP-IP)

A Magna RIP^TM^ RNA-Binding Protein immunoprecipitation kit (Cat. #: 17-700, Millipore, Merck KGaA, Darmstadt, Germany) was used following the manufacturer’s guidelines. For immunoprecipitation, primary antibodies against the following proteins were used: anti-AGO2 (Cat. #: ab57113, Abcam, Cambridge, UK) and anti-rabbit polyclonal IgG (Millipore, Merck KGaA, Darmstadt, Germany).

### Statistical analysis

Data are represented as the mean ± S.D. from triplicate independent experiments. Statistical analysis was performed using Student’s *t*-test. For assessment of gene expression and overall survival using publicly available datasets, the PROGmiR (http://www.compbio.iupui.edu/progmir) and PREdiction of Clinical Outcomes from Genomic Profiles (PRECOG) (https://precog.stanford.edu/) were used for analysis of microRNA and mRNA, respectively [45, 46]. The results were directly copied from these online tools with little modification. Difference was considered significant when p < 0.05.

## SUPPLEMENTARY MATERIALS FIGURES AND TABLES



## Abbreviations

APL: acute promyelocytic leukemia
ASO: Anti-Sense Oligonucleotide
ATO: Arsenic Trioxide
DCFDA: 2’, 7’ –dichlorofluorescin diacetate
GBM: glioblastoma multiforme
GEO: Gene expression omnibus
GO: gene ontology
GSEA: Gene set enrichment analysis
IPA: Ingenuity Pathway Analysis
MTT: 3-(4,5-dimethylthiazol-2-yl)-2,5-diphenyltetrazolium bromide
NAC: N-acetyl cysteine
RISC: RNA-induced silencing complex
RNP-IP: ribonucleoprotein immunoprecipitation
ROS: reactive oxygen species
UTR: untranslated region
