# Supplementary material for: Arsenic trioxide-mediated suppression of miR-182-5p is associated with potent anti-oxidant effects through up-regulation of SESN2
Source: Oncotarget. 2018 Mar 23;9(22):16028–42. doi: 10.18632/oncotarget.24678 (PMC5882315; doi:10.18632/oncotarget.24678)
Supplement: Supplementary file 1 [file oncotarget-09-16028-s001.pdf]

Arsenic trioxide-mediated suppression of mir-182-5p is associated with potent anti-oxidant effects through up-regulation of *SESN2*

SUPPLEMENTARY MATERIALS

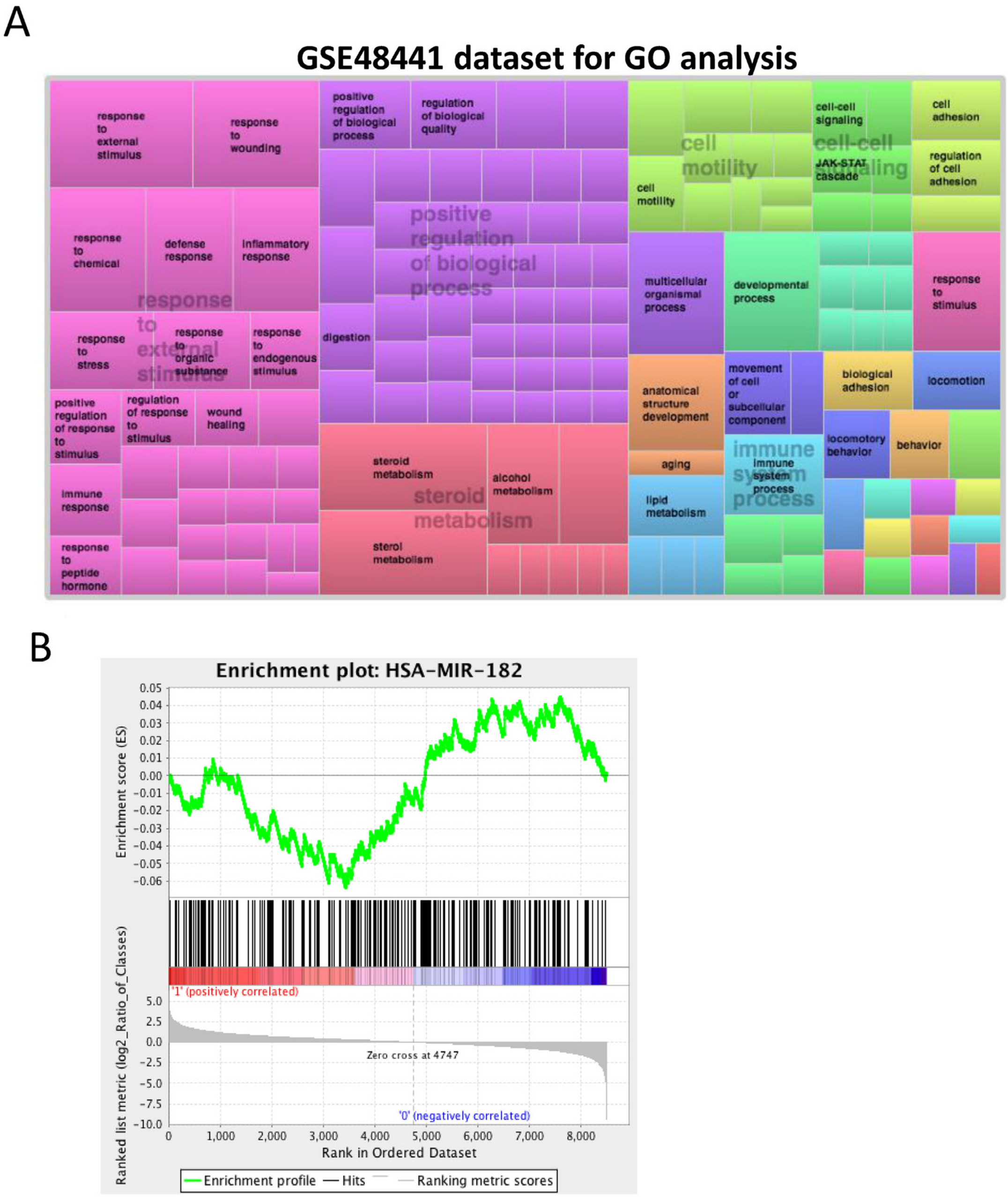

Supplementary Figure 1: Bioinformatics analysis of ATO-mediated changes in gene expression. (A) Microarray database from Gene Expression Omnibus (GEO, accession number GSE48441) was subjected to gene ontology (GO) analysis, and REVIGO webbased software was used to identify genes induced by 6 M ATO. Each color code represents a category of response related to corresponding gene expression profiles. The leftmost pink code represents "response to external stimulus, and the midst purple code represents "positive regulation of biological process", and so on. **(B)** Gene set enrichment analysis (GSEA) identified that miR-182-targeted genes are significantly enriched by treatment with a moderate concentration of ATO ($p<0.05$).

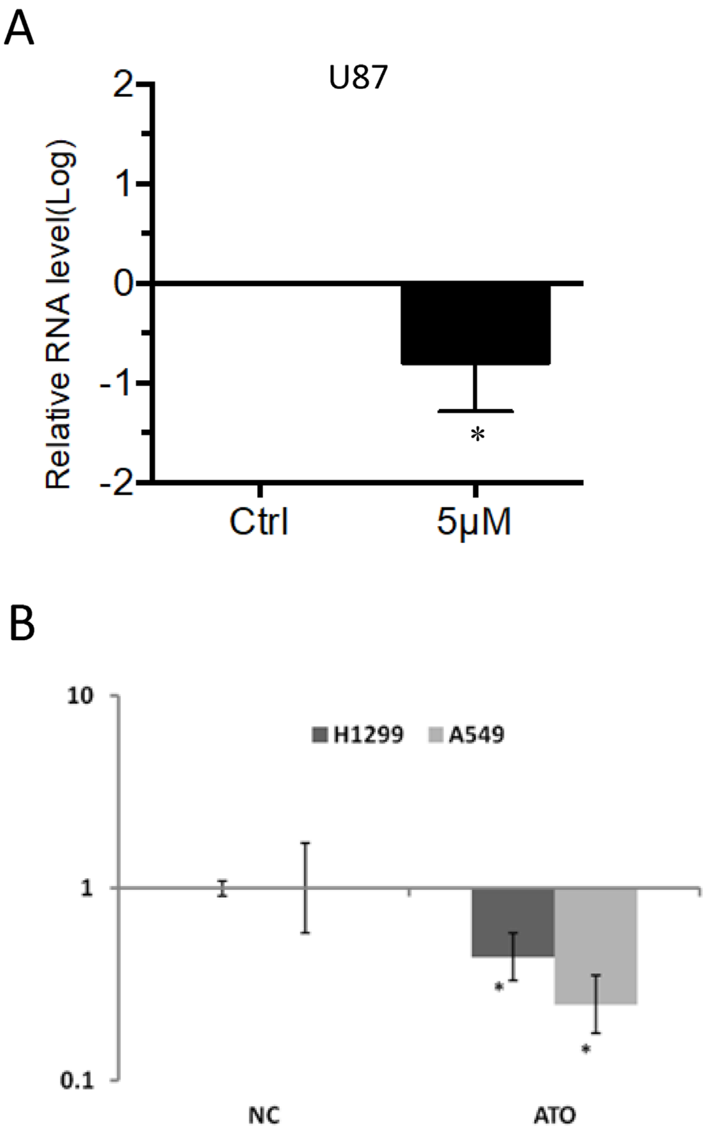

Supplementary Figure 2: The level of miR-182-5p of **(A)** U87MG cells, **(B)** H1299 cells and A549 cells before and after treatment of 5mM ATO. *: $p < 0.05$.

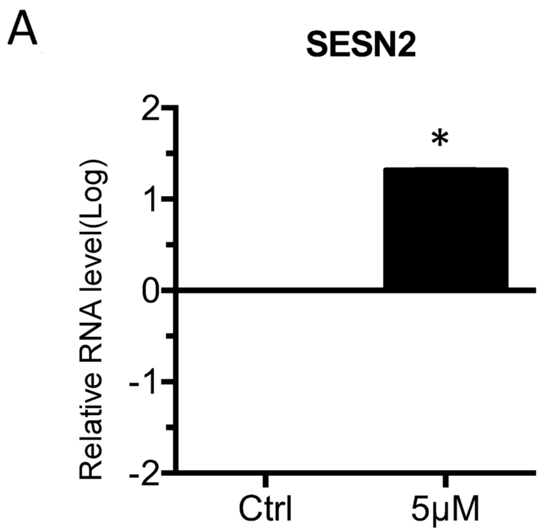

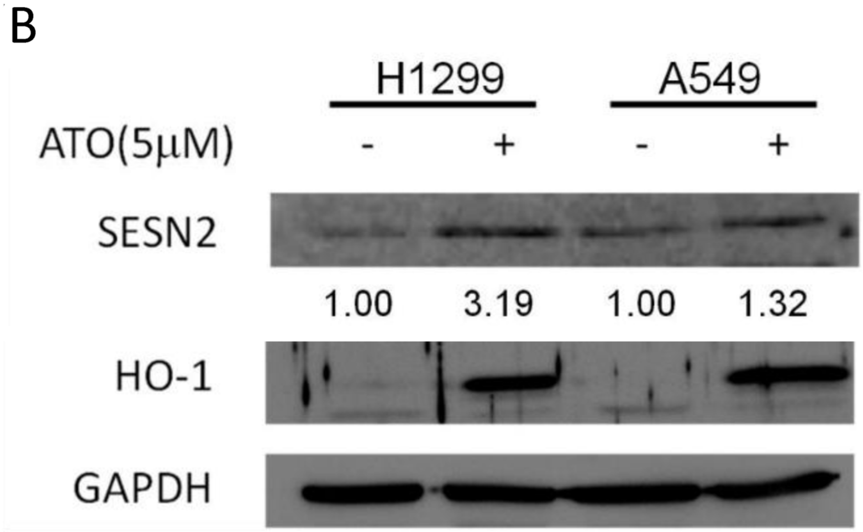

Supplementary Figure 3: (A) ATO induced expression of SESN2 mRNA in U87MG cells, **(B)** ATO induced expression of SESN2 protein in H1299 cells and A549 cells. *: $p<0.05$.

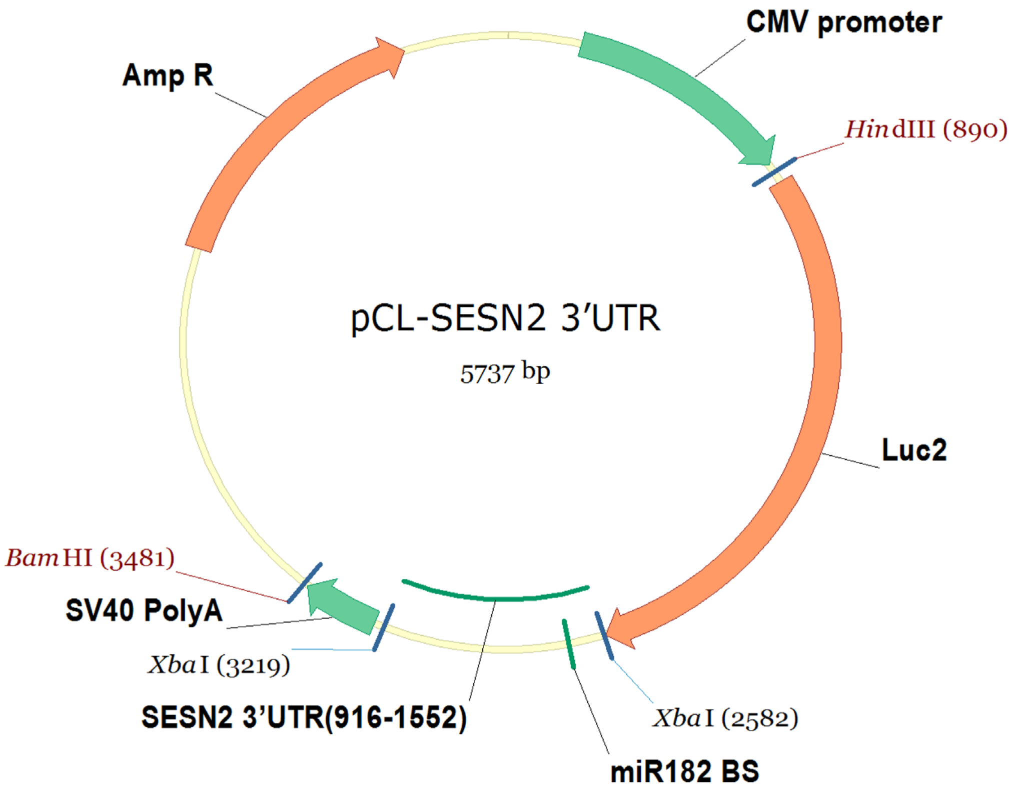

Supplementary Figure 4: The plasmid map of pGL4.10-Luc-SESN2 3'UTR vector
